# Limb Regeneration in Axolotl: Is It Superhealing?

**DOI:** 10.1100/tsw.2006.324

**Published:** 2006-05-05

**Authors:** Stéphane Roy, Mathieu Lévesque

**Affiliations:** Department of Stomatology, Faculty of Dentistry, Université Montréal, C.P. 6128 succursale Centre-ville Montréal, Que. H3C 3J7, Canada

**Keywords:** axolotl, urodele, limb, regeneration, wound healing, functional analysis, amphibians

## Abstract

The ability of axolotls to regenerate their limbs is almost legendary. In fact, urodeles such as the axolotl are the only vertebrates that can regenerate multiple structures like their limbs, jaws, tail, spinal cord, and skin (the list goes on) throughout their lives. It is therefore surprising to realize, although we have known of their regenerative potential for over 200 years, how little we understand the mechanisms behind this achievement of adult tissue morphogenesis. Many observations can be drawn between regeneration and other disciplines such as development and wound healing. In this review, we present new developments in functional analysis that will help to address the role of specific genes during the process of regeneration. We also present an analysis of the resemblance between wound healing and regeneration, and discuss whether axolotls are superhealers. A better understanding of these animals' regenerative capacity could lead to major benefits by providing regenerative medicine with directions on how to develop therapeutic approaches leading to regeneration in humans.

## INTRODUCTION

Regenerative medicine is an emerging field of medical research that promises to unlock the secrets of how the body can regenerate itself. The ability to regenerate lost or damaged tissues would greatly benefit people suffering from the loss of bodily function such as organ failure, amputation of a limb, or third-degree burns. Like any other new frontier of sciences, regenerative medicine will only flourish if we can understand the fundamental mechanisms and signaling pathways that can stimulate our bodies to heal themselves perfectly. Unfortunately, mammals are incapable of regenerating lost tissues and are unable to heal their wounds without scarring as adults, therefore making the traditional mammalian models (i.e., mouse and rat) less useful as the models of choice. At present, one of the main approaches to mammalian regenerative medicine is the isolation of stem cells, followed by manipulations that are aimed at directing their differentiation towards the morphogenesis of complex structures[1]. Although this attracts a significant amount of interest at present, many applications are of such complexity to warrant consideration of alternative and complementary approaches. Fortunately for tissue regeneration research, there is a group of vertebrates called *urodeles* that can perfectly regenerate various tissues following amputation or injury through the process of epimorphic regeneration[2,3,4,5]. The axolotl (*Ambystoma*

*mexicanum*) and the newt (*Notophthalmus viridescens*) are the two most commonly used urodele amphibians in the laboratory and both possess unsurpassed abilities to regenerate body parts (limbs, jaws, tail, spinal cord, etc.) and heal their wounds without scars throughout their life[5,6,7]. Urodeles are the only tetrapod vertebrate that can recreate the "embryonic" or "undifferentiated" environment for redevelopment to occur once cells have differentiated.

Among the complex structures that can perfectly regenerate in urodeles, the limb is the best described and studied. For the better part of the past century, the research on regeneration focused on unfolding the cellular events and the order in which the tissues get reorganized throughout regeneration. The origin of the cells giving rise to the regenerated tissues was resolved by these carefully designed experiments, which included grafting, irradiation, denervation, chemical treatments, etc. that spanned many decades of intense research. These studies were essential in providing us with our present-day understanding of the temporospatial sequence of events and the role played by tissue interactions that occur during regeneration. From the information generated by the aforementioned experiments, scientists were able to develop two major models to explain morphogenesis and how the exact replacement of the missing parts of the limb were possible: the polar coordinate model and the model of regeneration by intercalation[8,9,10,11]. With the advent of molecular biology, the field is now focusing on the identification of the genes and signaling pathways orchestrating the cellular events that give rise to the replacement of lost or damaged tissues. The characterization of the hierarchal cascade of genes important or essential for regeneration and/or perfect wound healing in urodeles is of interest for multiple disciplines of fundamental and biomedical research. A better characterization of the signaling pathways responsible for mediating the regenerative process is essential if we are to understand why mammals form scar tissue instead of regenerating perfectly. More importantly is the potential benefit that this field of research offers for biomedical research and eventually the development of novel medical treatments.

## DEVELOPMENT VS. REGENERATION

Limb regeneration is often referred to as a biphasic process that is comprised of a preparation phase and a redevelopment phase, each composed of several stages[3,12,13,14]. Multiple observations have led scientists to propose this biphasic process. The first phase represents the regeneration-specific phase where amputation (the trigger of regeneration) occurs, which is followed by the formation of a wound epithelium from migrating epidermal cells (which will later thicken to become the apical epidermal cap [AEC]), immune response[131], cellular dedifferentiation of multiple cell types132], proliferation, and migration of these dedifferentiated cells to give rise to the blastema[2,15]. Nerves are also essential for cellular migration and division during the first part of the regeneration process[5,16]. This preparation phase spans the initial stages of regeneration until the end of medium bud[3,5,17]. If any of these events are prevented, such as the formation of the wound epithelium, or the limb is denervated, regeneration is inhibited. The second phase represents the redevelopment phase of regeneration (Fig. 1); it spans from the late bud stage until the limb has completely regenerated. As in development, pattern formation/morphogenesis is established and nerves are no longer required for this phase. If denervation occurs at late bud or later, regeneration continues as if nothing happened and a normal limb appears[18,19,20,21]. The expression of multiple genes during the redevelopment phase of regeneration displays a pattern that is highly reminiscent of that observed during embryogenesis[3,22,23,24,25,26,27,28,29]. The expression of *sonic hedgehog* and the expression of multiple *hox* genes are basically identical during the redevelopment phase of regeneration as observed during limb development[22,23,28,29]. Gardiner et al. published an interesting paper where they described the expression pattern of *hox A9* and *hox A13* during axolotl limb development and regeneration. They demonstrated that the expression of these two *hox* genes is conserved during limb development where *hox A9* is expressed early in the entire limb bud and *hox A13* is expressed later in the distal-most part of the limb bud as in other vertebrates[23]. The proximal-distal expression of these genes during regeneration is quite different during the preparation phase of regeneration where both genes are

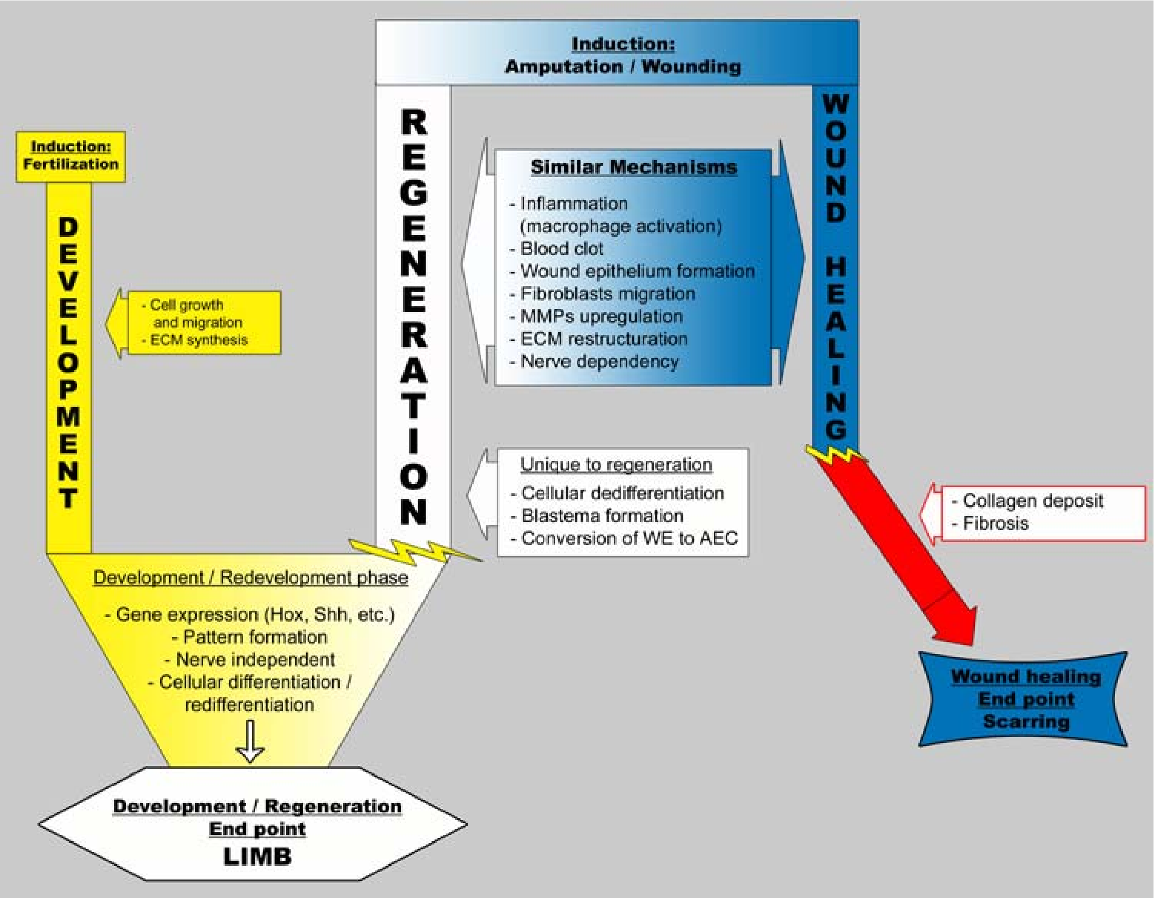

**FIGURE 1.** Parallels between development, wound healing, and regeneration. Regeneration is a biphasic phenomenon with a preparation phase and a redevelopment phase. The preparation phase displays many mechanisms that share similarities to those observed during the earlier part of wound healing. The redevelopment phase, as its name indicates, shares many similarities with development. The appearance of the AEC, cellular dedifferentiation, and blastema formation represent processes that are entirely unique to regeneration.

expressed early on in an overlapping pattern. As regeneration progresses, the pattern of expression for *hox A9* and *hox A13* starts to separate such that once in the redevelopment phase, *hox A9* is expressed in the entire blastema and *hox A13* is expressed in the distal part of the blastema, which is similar to development. Interestingly, genetic studies have linked the overlapping expression of *hox A9* and *hox A13* with the patterning of the most distal part of the limb, whereas more proximal regions would express *hoxA9*, but not *hoxA13*[30]. These results are in perfect agreement with the model of regeneration by intercalation, which was initially demonstrated by grafting a distal blastema onto a proximal stump, which resulted in a normal regenerate and vice versa that stipulates that the most distal part of the limb is specified first and the sections between the stump and blastema are intercalated after[10,11]. Of course, not every developmental gene that has been studied in regenerating limbs presents such a perfectly fitting expression pattern with the preparation and redevelopment phase. For example, the expressions of fibroblast growth factor (*fgf*) genes do not all display expression patterns during regeneration that fit with the model of regeneration by intercalation (e.g., *fgf-4* and *fgf-10*)[31,32]. *Fgf-8*, on the other hand, does display a pattern of expression during regeneration that correlates more, although not perfectly, with the model of regeneration by intercalation. It is up-regulated at medium and late bud stages, which represent the beginning of the redevelopment phase, and is down-regulated at palette when the morphogenesis of the fingers becomes apparent, which is similar to the expression pattern observed during axolotl limb development[33]. The number of genes that has been studied in urodele limb regeneration is constantly increasing and, therefore, we will limit our analysis of gene expression to the few mentioned above, which is by no means exhaustive.

The parallels between development and regeneration are to be expected, at least for a number of genes, since the end result, in our case, for both processes is the formation of a limb. There is no reason to expect a whole new set of genes just for morphogenesis during regeneration when the organism can use its developmental genes. In fact, regeneration is most likely the result of the ability of urodeles to recruit or call into play all the necessary genes and signaling molecules in the proper order (i.e., hierarchal cascade) in response to an injury/amputation rather than being the only vertebrates that evolved regeneration specific genes.

## NERVES

The requirement of proper innervation during regeneration is a paradigm that has always generated interest[5]. Interestingly, all systems that have some regenerative capacity in other vertebrates (e.g., liver regeneration, scarless fetal wound healing, and bone healing) also require proper innervation[34,35,36]. Nerves are, therefore, either an essential component or are responsible for releasing one or more molecules of paramount importance to achieve any level of regeneration. The urodele regenerating limb offers a unique opportunity to identify nerve factors that contribute to this dependency. It is very easy to denervate the urodele forelimbs since only three superficial large nerves supply all the innervation[37], unlike the other systems mentioned above. In a study by Mullen et al., it was shown that *Dlx-3* was down-regulated following forelimb denervation during the preparation phase of regeneration[18]. *Dlx-3* is a homeobox transcription factor involved in epithelial/mesenchymal interactions. These interactions are essential for the development of multiple structures (e.g., teeth, limbs, kidneys, lungs) in vertebrates[38,39,40,41,42,43,44]. It seems that in the regenerating limb, nerves are important mediators of these epithelial/mesenchymal interactions that occur between the blastema cells and the AEC[5,18]. The importance of these epithelial/mesenchymal interactions is supported by the fact that preventing the formation of the wound epithelium (either by removing it, closing the open wound with stitches, or a mature skin graft) inhibits regeneration[20,45]. Additional studies will be necessary to better understand the requirement of nerves and the epithelial/mesenchymal interactions and to identify the genes responsible for mediating these interactions in the context of regeneration.

## FUNCTIONAL ANALYSIS

The study of regeneration in urodeles suffered for many years from the lack of tools that would allow investigators to perform functional analyses of the genes expressed during the different phases of regeneration. Over the past 25 years, multiple approaches have been developed to achieve functional analysis in a variety of model organisms. One of the most prominent techniques for functional analysis is, by far, the development of transgenic and knockout mice. This allows investigators to manipulate any desired gene that can be ectopically expressed or deleted. This ability has provided an unprecedented view into the molecular mechanisms regulating developmental processes in vertebrates. Unfortunately, this methodology is more difficult to apply in axolotls due to the slow sexual maturity of these animals (12–18 months required for reproduction) and the lack of knowledge of their genome. Finally, the use of embryonic stem cells to generate knockout animals has not been achieved in any other organisms beside mice. Still, with the great interest in developing novel and efficient tools for gene therapy in humans, we now have access to many different approaches that show promise for gene delivery such as liposomes, viral vectors, and biolistic-, sonoporation-, and electroporation-mediated gene delivery directly into tissues.

The vaccinia virus was the first viral vector to be used successfully to test the function of a specific gene of developmental interest during regeneration in axolotls[27]. The ectopic expression of *shh*, delivered with vaccinia, in the anterior region of the blastema resulted in supernumary limbs[27]. The overexpression of *shh* caused polydactyly only at the stages of regeneration when endogenous *shh* is expressed in the posterior region of the blastema[27]. This was the first functional study to address specifically the role of an important developmental gene expressed during regeneration. It was also the first demonstration that axolotls are amenable to functional analysis and that viral vectors can achieve close to a 100% infection efficiency of the blastema cells[27]. Lentiviral vectors have also been shown to infect and drive the expression of the reporter gene beta-galactosidase in axolotl cells both *in vitro* and *in vivo*[46]. Their ability to drive the expression of functional genes has yet to be demonstrated in axolotls, but one can presume that they will be able to lead to the functional expression of genes as in other species. Some studies have also been performed using biolistic delivery of gold particles impregnated with plasmids resulting in a transfection rate of 10% for epidermal cells. That study demonstrated, for the first time, that this method can be used for functional analysis of nuclear receptors in urodeles[47].

Electroporation has become a method of choice for transfecting plasmids into chicken embryos[48,49,50,51,52,53,54,55]. It presents some advantages over viral vectors in that they are easier to generate, they have no infectious potential, and expression of the ectopic gene is very rapid. This technique has recently been adapted to the axolotl’s regenerating tissues by Tanaka’s research group who succeeded in performing functional analysis studies for limb and tail regeneration[56,57,58,59,60]. They were not only able to overexpress genes, but were also able to inhibit the expression of specific genes by combining the use of antisense morpholinos with electroporation[59]. This represents an important technical development, since it allows investigators to perform both gain and loss of function studies.

The most significant and exciting new development in terms of performing functional analysis studies in urodele amphibians has been the ability to develop and raise transgenic salamanders[61,62]. Both Japanese newts (*Cynops pyrrhogaster*) and axolotls were shown to be amenable to transgenesis[61,62]. Two different approaches were used to achieve transgenesis for each salamander. The transgenic Japanese newts were generated by mixing the linearized transgene DNA with permeabilized sperm nuclei, isolated from fresh testis as is done for *Xenopus laevis* transgenesis[63], and then injected into unfertilized eggs with inositol 1,4,5-triphosphate[62]. Transgenic axolotls were generated by using the I-Sce1 meganuclease method, which is used to generate transgenic fishes and frogs[64,65]. Transgene DNA, flanked by the Sce1 sites, was coinjected with Sce1 nuclease into fertilized axolotl eggs before the first cell division[61]. Both methods resulted in the expression of the reporter gene, green fluorescent protein, in all the tissues observed. However, no germ line transmission was reported in the newt as opposed to the observed germ line transmission in axolotls[61,62]. These exciting results are opening the door to more thorough investigation into the specific roles of genes during regeneration. One can envisage the combined use of generating a transgenic salamander that overexpresses cDNAs to increase protein expression or short hairpin RNAs to specifically inhibit gene expression. Inducible promoters, such as the tetracycline promoter, could also be incorporated into the transgene DNA construct to control the exact time of induction. Tissue-specific promoters have already been shown to work in salamanders, even if they are from another vertebrate species, therefore eliminating the need to clone all the tissue-specific promoters of the axolotl to achieve this goal[62]. These new technical developments in urodele transgenesis are making it possible for the first time, since scientists have marveled at the ability of salamanders to regenerate their tissues[66], to answer questions pertaining to the functional roles of specific genes during the various phases of regeneration.

The pharmacological approach also represents another means of achieving functional analysis in regenerating tissues. One just has to think of the large body of literature published over the past 40 years on the effects of retinoic acid on regeneration and pattern formation of the regenerating limb of salamanders[67,68,69,70,71,72,73,74,75]. It provided important information on the positional values present in tissues and it also helped give rise to the theory of polar coordinate model by French and Bryant[9]. Another important pharmacological compound that has more recently been very useful to understand the functional role of *shh* during regeneration is cyclopamine. Cyclopamine is a naturally occurring compound isolated from the plant skunk cabbage (*Veratrum californicum)* and is a potent and specific inhibitor of hedgehog signaling[76,77,78,79,80,81,82]. When axolotls were treated with cyclopamine during limb regeneration, it resulted in the formation of cartilaginous spikes where complete loss of anterior/posterior patterning was observed[26]. This phenotype corresponds to the limb phenotypes observed in the *shh* knockout mouse[83,84]. At present, the pharmacological approach is limited in the number of drugs that target specific genes or signaling cascades. However, with the ever-increasing need to develop more efficient drugs to treat various pathologies and the development of combinatorial chemistry, which is giving rise to many new classes of small molecules, there is reason to expect that more and more compounds will be available. Combinatorial chemistry (combichem, combinatory chemistry) is a technique by which large numbers of structurally distinct molecules may be synthesized simultaneously and submitted to pharmacological assays. Some of these new compounds display specificity for certain enzymes (e.g., GM6001 inhibitor of mmps; SB 203580 a p38 MAP kinase inhibitor, just to mention a few) and we can expect to have more compounds in the years to come. This will be of great use for assessing the role of various signaling pathways during regeneration. Some novel molecules, like myoseverin and reversine, are already helping us to understand certain aspects of cellular changes observed during regeneration[85,86,87,88,89,90,91,92,93,94,95,96]. There are obvious advantages to using pharmacological agents in that they are easy to administer (simply add to the water that the axolotls are kept in), all the cells are exposed, effects are often immediate as well as reversible, and they can be purchased from the manufacturers. Of course, some of these advantages can also be considered disadvantages, for example, if one wants to treat exclusively epidermal cells.

## REGENERATION VS. HEALING?

Over the past decade, multiple review articles have put emphasis on the differences and similarities between development and regeneration, which have led scientists to propose that regeneration is a biphasic phenomenon as mentioned earlier[2,12,13,14]. The comparison is justified since the end result is the formation of the same structure either during development or regeneration using, in large part, the same molecular machinery[25]. However, it can also be argued that this comparison is partly due to the bias of developmental biologists like us who, for the most part, are the ones studying limb regeneration. If one considers the immediate medical applications or the most common problems faced in a clinical setting that would benefit from a better understanding of regeneration, we are brought to think about scarring/fibrosis as being the problem of central importance for the general population. Scarring is obviously the result of wounding, which can be either accidental or as a result of surgery. Fibrosis can also be of pathological origins, as in scleroderma[97]. It is interesting, therefore, to highlight the fact that following limb amputation, the axolotl will regenerate its limb without any residual scar between the stump and the regenerated structure; it is perfect. It is noteworthy to mention that the vast majority of vertebrates including mammals, birds, anuran amphibians, and reptiles are incapable of regenerating complex structures such as limbs. Thus, scarring or fibrosis seems to be a universal response in dealing with wounds that span the three layers of the skin in vertebrates. Even the sea cucumber, which has the amazing capacity to regenerate a large portion of its viscera, is incapable of healing its skin wounds perfectly[98]. If we compare wound healing in the most studied group of vertebrates, mammals (mice and humans), vs. urodeles, we find that scar tissue formation is not easily observed in axolotls following excisional or incisional wounds (personal observation). Actually, axolotls will heal their skin wounds without the appearance/formation of scars[6]. This brings us to consider the similarities between regeneration and wound healing, which can help us better understand how regeneration is achieved in urodeles but not in mammals. Fig. 1 illustrates the similarities between regeneration and development (as depicted in other reviews) and also between regeneration and wound healing (as observed in postnatal and adult mammals). Interestingly, the similarities between regeneration with development and with wound healing correspond to the different phases of regeneration. The similarities with respect to development reside in the second phase of regeneration (also referred to as the redevelopment phase)[2,3,12,13,14]. Similarities between regeneration and wound healing, on the other hand, correspond to the preparation phase of regeneration. One of the first similarities that can be pointed out is the induction signal that consists of a trauma (amputation in one case and a wound that spans the three dermal layers of the skin in the other). Many stress signals are activated by the trauma, which is followed by inflammation (which triggers macrophages activation) and formation of a blood clot. These events all occur within minutes of the amputation/wound and are followed shortly by the formation of a wound epithelium (WE), which for both regeneration and wound healing is essential for either of these processes to proceed to the next phase[5,99,100,101]. The formation of a WE is, in fact, one of the first observable steps to occur in limb regeneration and wound healing; it is usually formed within 2–6 h postamputation in urodeles and between 12 h to 2 days postwounding in mammals[102,103]. If formation of the WE is prevented, regeneration does not proceed and wound healing is retarded significantly. The WE is indispensable for the establishment of epithelial/mesenchymal interactions as mentioned above. These interactions are essential for epimorphic regeneration and wound healing, as they are for multiple aspects of development[38,39,40,41,42,43,44,104]. After the WE is formed, extracellular matrix (ECM) restructuring is initiated by the up-regulation of matrix metalloproteinases[103,105,106,107,108]. The ECM is remodeled extensively during limb regeneration (as are the muscles, blood vessels, nerves, and bones at the amputation site) through the action of the same matrix metalloproteinases (*mmp*) and tissue inhibitors of metalloproteinases as those for wound healing in mammals[109,110]. *MMP*-2, -9, and -13 are expressed during regeneration and skin wound healing. Inhibitors of *mmp*s have been shown to inhibit limb regeneration and to cause the appearance of a scar-like layer of skin with collagen deposits on the stump of amputated limbs treated with GM6001, a *mmp* inhibitor[107].

Soon after ECM restructuring has begun, dermal fibroblasts start to migrate under the WE and epithelial/mesenchymal interactions are established. Fibroblasts in wound healing will give rise to granulation tissue[111], whereas in limb regeneration, they will contribute to form the blastema[5,112]. Both epimorphic regeneration and wound healing require a proper nerve supply[2,18,21,113,114,115, 116,117].

During limb regeneration, muscle cells also play a role in the formation of the regeneration blastema and, like the dermal fibroblast, they are generated by a process of cellular dedifferentiation followed by high levels of proliferation[132]. Cellular dedifferentiation and blastema formation represent major cellular events unique to regeneration and not observed in wound healing.

Fibroblasts are essential for both processes as they provide cells to generate the missing tissues. Obviously during skin wound healing, muscle cells do not play a role since the experimental wounds usually only span the dermal layers and do not reach the muscles underneath. Interestingly, dermal fibroblasts are sufficient to give rise to an entire limb, except for muscles, as demonstrated by grafting an intact skin cuff onto an irradiated limb[118,119]. The importance of dermal fibroblasts has also been shown in ectopic limb regeneration on the upper arm of the axolotl[120]. To generate these ectopic limbs, a nerve is deviated and a contralateral skin graft is placed to create a positional confrontation between two pieces of skin corresponding to different positions as per the polar coordinate model[9]. If the skin graft does not contain dermal fibroblasts, no ectopic limb will regenerate/grow[120].

## IS REGENERATION IN AXOLOTLS SUPERHEALING?

From the schematic representation of the similarities between limb regeneration and wound healing presented in Fig. 1, it is reasonable to ask whether regeneration is basically superhealing, which urodeles have been able to master. This is, of course, an easier question to ask than it is to answer. It is not easy to design an experiment that would demonstrate directly whether regeneration is, in fact, a heightened capacity to heal. There is one experiment, however, that has led us to hypothesize that limb regeneration is not a superhealing phenomenon. Fig. 2 shows the results observed in axolotls following bone fracture healing as well as the healing of nonunion fractures surpassing the critical gap size for bone healing to occur[121,122,123]. Bone fractures with a gap between the two extremities larger than a certain size (gap size varies with the size of the animal) will not heal and are therefore referred to as gaps of critical

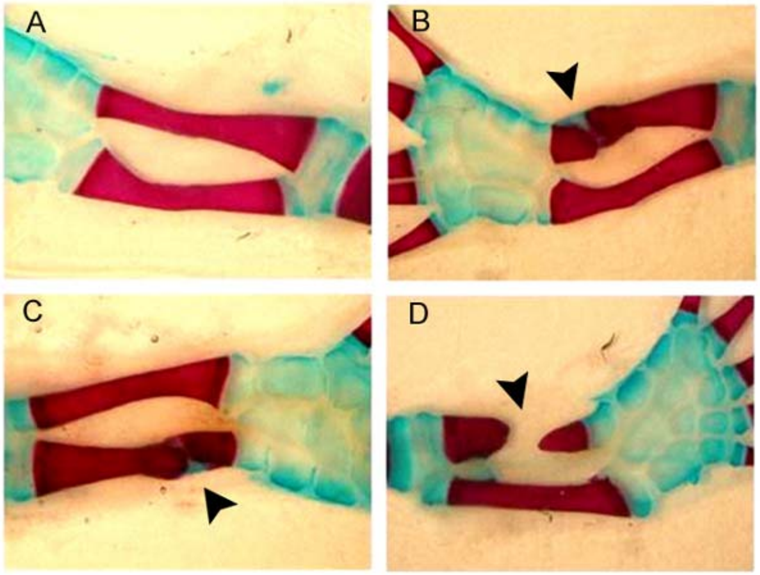

**FIGURE 2.** Double staining of axolotl limbs, with alizarin red (bone) and alcian blue (cartilage). (A) Control limb, sham no fracture; (B and C) depicting bone healing of the ulna for nonstabilized fractures with a callus formed of cartilage (black arrows); (D) gap of critical dimension (4 mm) with no signs of healing (black arrow). All pictures were taken 5 months postfracture.

dimension[122]. We can see that simple fractures in nonstabilized limbs heal by forming a callus via a cartilaginous phase (Fig. 2 B and C in blue). Fig. 2D depicts the result of a gap of critical dimension unable to heal even 5 months postfracture. If the axolotl's ability to regenerate was simply an extension of healing, i.e., superhealing, then one would expect them to be able to heal critical gap fractures. However, it could be argued that the surrounding tissues inhibit the gap from healing by preventing bone cells from migrating to the proper position. Some may be tempted to propose that regeneration needs exposure to the outside environment, although the apex of the heart and the spinal cord can regenerate without such exposure and in the presence of surrounding tissues. What is particularly interesting and represents somewhat of a paradox is that bones are completely regenerated if a limb is amputated[5,17,124,125]. The source of cells giving rise to new bones is not likely to be from the bones in the stump[112,118]. Two series of experiments lend their support to this assertion: first, the rescue of regeneration in irradiated limbs with a graft of healthy skin where all the tissues, except the muscles, are present in the regenerate; and second, the extirpation of the ulna followed by amputation of the limb results in a regenerated limb with a partially regenerated ulna, of the proper dimension and at the proper place, in the newly regenerated portion of the limb, as if the ulna was left in place in the stump[124]. These experiments indicate that in order to regenerate new skeletal elements, material from the elements present in the stump is not needed. Irradiation causes DNA breaks and prevents cells from dividing[119,126], therefore all the cells that constitute the newly regenerated limbs have to be derived from the graft of healthy skin[118]. The appearance of a partial ulna, properly positioned, in regenerating limbs in which the ulna had been previously extirpated suggests that regenerating bones do not need the corresponding bones in the stump for initiating skeletogenesis or positioning. The latter results are consistent with the polar coordinate model, suggesting that the skin or dermis contains all the necessary positional information for the newly regenerated tissues[9,127]. The results in Fig. 2D are also somewhat consistent with the regeneration by intercalation model that requires a blastema in contact with the stump for epimorphic regeneration to take place[10,11]. If a mature hand is grafted onto a proximal stump nothing happens (i.e., no intercalation), but if a distal blastema is grafted onto a proximal stump, a normal limb will regenerate with no missing parts. The critical gap fractures resemble the grafting of a mature hand onto a stump in the experiments of regeneration by intercalation where no intercalation of missing structures takes place. Of course, this may be due to the fact that a mature hand does not have an AEC that is permissive and, hence, no epithelial/mesenchymal interactions can take place. We can draw a resemblance between wound healing and the preparation phase of regeneration in terms of a certain number of cellular changes (see Fig. 1) and the expression of some genes common to both processes. However, we cannot say that regeneration is simply an improved version of wound healing based on the results observed in irradiated limbs, the regeneration of a partial ulna, induction of an ectopic limb, and the inability to heal gaps of critical dimensions. Mescher et al.[131] present an interesting analysis of the immune response in relation to epimorphic regeneration in which they present convincing data that implicate a diminished immunological or, more precisely, an immunotolerance response in urodeles following amputation. Their immunological considerations support the idea that regeneration is not superhealing, but a rather different response, related to immunotolerance, of urodeles to injury that may be in part responsible for directing or allowing cells at the amputation site to proceed towards regeneration.

## WHAT'S NEXT?

The past 10 years have seen remarkable new advances in the use and development of the axolotl as a model organism to study the cellular and molecular mechanisms responsible for driving epimorphic regeneration in vertebrates. The next development that we, as the scientific community working on regeneration, would certainly like to witness in the near future is the development of axolotl microarrays. EST sequencing projects in the U.S. and in Germany[128,129] represent the first steps towards the realization of microarrays. Once we have this powerful tool in hand, it will be possible to tackle questions regarding the identification of genes involved in cellular dedifferentiation, nerve dependency, cellular redifferentiation, and the molecular characterization of the switch from preparation phase to redevelopment. The possibility to finally be able to identify, on a large scale, the genes implicated in the various aspects of regeneration is important in providing the knowledge to target the genes on which we should focus our functional analysis studies (see section above).

Genomic studies have consistently shown that developmentally important genes are strongly conserved across vast evolutionary distances. Much of what we know of vertebrate development and molecular genetics has been derived from studies on *Drosophila* and other invertebrates. Interestingly, parallels between tissue repair in *Drosophila* and mammals have led others to state that the fruit fly embryo is as good a model for wound healing as the mammalian embryo[130]. Therefore, it is not unreasonable to expect that the same genes involved in the limb regeneration of amphibians are conserved in humans, though their activity may be either suppressed or neutralized by inhibitory factors in humans or enhanced in amphibians. Thus, based on our knowledge that genes and their functions are highly conserved between species, the idea of using the axolotl, which is unique in its regenerative capacity, to uncover the mechanisms leading to perfect wound healing and regeneration in mammals is an excellent approach if we are to understand how complex structures in vertebrates can be regenerated from our own cells.

## ACKNOWLEDGMENTS

The authors would like to thank members of S. Roy's laboratory and Dr. Jean Barbeau from the Faculty of Dentistry at the Université de Montréal, for their critical review of this manuscript. Regrettably, we could not refer to all the pioneering work that has been published on this subject due to the limited scope of this review; thus we apologize to all the authors whose work we did not cite. This research is supported by grants from the Canadian Institute of Health Research, the National Science and Engineering Research Council of Canada, and the Fonds de la Recherche en Santé du Québec. M.L. is supported by a Ph.D. scholarship from the Réseau de Recherche en Santé Buccodentaire du Québec.

## BIOSKETCHES

**S. Roy** obtained his PhD. in biology from McGill University in Montreal, under the supervision of Dr. H.S. Tenenhouse. He then joined the laboratory of Dr. S.V. Bryant at the University of California Irvine for a 3-year postdoctoral fellowship, where he acquired expertise in tissue regeneration using the axolotl as a model organism. During this first post-doc, he was successful in adapting the vaccinia viral vector in order to perform, for the first time, functional analyses of secreted proteins in regenerating limbs of axolotls. He also did a second post-doc in the laboratory of Dr. M Gascon-Barré on the molecular mechanisms of liver regeneration in rats. He has been assistant professor in the Department of Stomatology at the Université de Montréal since August 2001, where he is the director of a laboratory studying the molecular mechanisms involved in limb regeneration using the axolotl as a model organism.

**M. Lévesque** is a Ph.D. student in the Department of Biochemistry under the supervision of Dr. Roy. He is the recipient of multiple scholarships and has authored multiple abstracts and papers on the subject of limb regeneration.
